# Attenuated iron stress and oxidative stress may participate in anti-seizure and neuroprotective roles of xenon in pentylenetetrazole-induced epileptogenesis

**DOI:** 10.3389/fncel.2022.1007458

**Published:** 2022-11-17

**Authors:** Mengdi Zhang, Yao Cheng, Yujie Zhai, Yi Yuan, Haoran Hu, Xianfeng Meng, Xuemeng Fan, Hongliu Sun, Shucui Li

**Affiliations:** School of Pharmaceutical Sciences, Binzhou Medical University, Yantai, China

**Keywords:** epileptogenesis, neuronal injury, oxidative stress, iron stress, xenon

## Abstract

The previous studies have demonstrated the excellent neuroprotective effects of xenon. In this study, we verified the anti-seizure and neuroprotective roles of xenon in epileptogenesis and evaluated the involvement of oxidative stress and iron accumulation in the protective roles of xenon. Epileptogenesis was induced by pentylenetetrazole (PTZ) treatment in Sprague-Dawley rats. During epileptogenesis, we found increased levels of iron and oxidative stress accompanied by elevated levels of divalent metal transporter protein 1 and iron regulatory protein 1, which are closely associated with iron accumulation. Meanwhile, the levels of autophagy and mitophagy increased, alongside significant neuronal damage and cognitive deficits. Xenon treatment reversed these effects: oxidative stress and iron stress were reduced, neuronal injury and seizure severity were attenuated, and learning and memory deficits were improved. Thus, our results confirmed the neuroprotective and anti-seizure effects of xenon treatment in PTZ-induced epileptogenesis. The reduction in oxidative and iron stress may be the main mechanisms underlying xenon treatment. Thus, this study provides a potential intervention strategy for epileptogenesis.

## Introduction

Epilepsy is a chronic neurological disorder caused by abnormal neuronal discharges in the brain and is accompanied by recurrent spontaneous seizures (Dhir, 2012; Lignani et al., 2020). It has a high impact, with a global prevalence of 0.5–1% and lifetime incidence of 1–3% (Fauser and Tumani, 2017). Epilepsy can cause neuronal damage and cognitive deficits. Although many drugs exist for the clinical treatment of patients with epilepsy, drug resistance and serious side effects are non-negligible (Schmidt and Loscher, 2005). Considering the unsatisfactory therapeutic effect of epilepsy, it is particularly important to explore potential protective strategies to prevent epileptic development.

Xenon is an inert gas that has been used in clinical practice as an anesthetic for more than 70 years (Maze and Laitio, 2020). Moreover, the safety of xenon has been verified through long-term clinical applications (Kulikov et al., 2019). In recent years, studies have demonstrated the excellent neuroprotective effects of xenon at sub-anesthetic doses (Metaxa et al., 2014; Yang et al., 2014; Lavaur et al., 2016a). For example, xenon promotes neuroprotection and repair by regulating glutamate metabolism in the cell culture and animal models of Alzheimer’s disease (Lavaur et al., 2017). In addition, remarkable neuroprotective effects have been demonstrated in models of local and global ischemia and spinal cord ischemia/reperfusion (Cattano et al., 2011; Yang et al., 2012; Metaxa et al., 2014; Yang et al., 2014).

Neuronal damage and synchronous discharge are characteristics of epilepsy and epileptogenesis (During and Spencer, 1993; Chiu et al., 2016; Kim and Kang, 2018). Xenon can inhibit synchronous neuronal firing (Uchida et al., 2012) and reduce neuronal damage caused by hyperexcitability by regulating glutamate metabolism (Lavaur et al., 2016a,b). Our previous studies demonstrated that xenon can inhibit seizures, neuronal damage, and cognitive deficits in kainic acid-induced status epilepticus and hypoxia-induced seizures (Zhang et al., 2019; Zhang M. et al., 2020; Zhu et al., 2020). Our previous findings and results of other studies suggest that xenon may exert anti-seizure and neuroprotective effects during epileptogenesis.

Normal regulation of iron metabolism, including iron intake, storage, and transport, is essential for brain development and function (Chen et al., 2019). Disturbances in iron regulation are closely associated with neuronal damage and can lead to degenerative diseases (Chen et al., 2019). For example, when excess iron is ingested and little iron is excreted, iron accumulation exerts toxic effects (Allen et al., 2018) and predisposes patients to diseases such as epilepsy and Parkinson’s disease (Jiang et al., 2010; Ayton et al., 2014).

Activation of the *N*-methyl-*D*-aspartate (NMDA) receptor critically contributes to epileptic hyperexcitability and hyperexcitability-induced neuronal injury. This activation can elevate the expression of divalent metal transporter protein 1 (DMT1), which is responsible for iron uptake and accumulation, and consequently leads to iron stress-induced apoptosis and neuronal injury (Cesar et al., 2012).

Additionally, the mutually reinforcing effects of iron stress and oxidative stress have been verified (Molinari et al., 2019). High iron levels can lead to oxidative stress through the Fenton reaction, and elevated levels of oxidative stress can further promote iron accumulation (Xu et al., 2008; Maremonti et al., 2020; Zhang M. et al., 2020). Hyperexcitability-induced oxidative stress is one of the main features of epilepsy and seizures (dos Santos et al., 2011). Thus, the simultaneous elevation of iron levels and oxidative stress caused by NMDA-regulated hyperexcitability may be associated with epileptogenesis and neuronal damage during this progression.

Xenon, an NMDA receptor antagonist, can also inhibit glutamate metabolism and eventually reduce hyperexcitability-induced damage (Lavaur et al., 2016a,b). Therefore, we hypothesized that xenon can inhibit seizures and neuronal injury during epileptogenesis by attenuating iron stress and oxidative stress.

We used the pentylenetetrazole (PTZ) kindling model to assess the differences in iron levels, oxidative stress, neuronal damage, cognitive deficits, and epileptogenesis between xenon-treated and non-xenon-treated rats to elucidate the protective effects of xenon and lay the foundation for future clinical applications.

## Materials and methods

### Animals

A total of 160 male Sprague–Dawley rats (220–250 g, 6-week old, no. SCXK2019-0003, provided by the Pengyue Experimental Animal Center, Jinan, China) were used in our experiments. The experiments were conducted in compliance with the National Institutes of Health Guide for the Care and Use of Laboratory Animals (NIH Publications No. 80-23, revised 1996) and ethical principles of the Binzhou Medical University Animal Experimentation Committee (approval no. 2020002). Each rat was housed in a separate cage. All animals had free access to food and water under a 12-h light-dark cycle. Efforts were made to reduce the number of animals and subsequent pain. All experiments were carried out between 8:00 and 17:00.

### Pentylenetetrazole kindling

The rats were intraperitoneally injected with PTZ (40 mg/kg, 10 mg/ml, CAS 54-95-5, Sigma-Aldrich, Saint Louis, MA, USA) once every other day for a total of 15 days. The control group was injected with an equal volume of saline. Each rat was individually placed inside a clear resin for 2 h for behavioral observation and electroencephalography (EEG) recording (ADInstruments, Dunedin, New Zealand). According to Racine’s seizure behavior standards (Racine, 1972), seizures were classified into stages 1–5.

### Xenon intervention

The xenon mixture (70% xenon, 21% oxygen, and 9% nitrogen) was introduced into a closed transparent resin-viewing chamber with an air inlet at the bottom and an air outlet at the top for 15 min at a controlled flow rate of 200 ml/min. The rats in the xenon group were then placed in the chamber and treated with the xenon mixture for 30 min immediately after the administration of each intraperitoneal PTZ injection. In total, there were 8 xenon treatments. The control group was treated with 21% oxygen and 79% nitrogen. After each PTZ injection, behavioral observations and EEG recordings were performed for 2 h.

Xenon treatment in our study was not sufficient to induce anesthesia, and the rats remained awake and were allowed to move freely throughout the experiment.

### Cognitive and behavioral testing

The Morris water maze (XR-Xmaze; Xinruan, Shanghai, China) experiment was performed 24 h after the eighth PTZ injection (Day 16) to assess the learning and memory abilities of the rats (Morris, 1984). The rat water maze device consisted of a circular pool (150 cm in diameter and 60 cm in height), station (12 cm in diameter and 20–35 cm in height), and a camera device. The detection was persisted for 5 days and was divided into two sections: positioning navigation experiment (Days 1–4) and space exploration experiment (Day 5). During the positioning navigation experiment, the rats were allowed to swimming for 60 s in order to find the platform that was hidden under the water (Zhang et al., 2019). If the rats failed to find the platform, a tester would enable the rats to find and remain on the platform for 10 s. As previously described (Zhang M. et al., 2020), the learning and memory abilities of rats in each group were assessed by detecting the latency to find the platform, cumulative number of passages over the platform, and percentage of time spent in the target and alignment quadrant (Netto et al., 1993; Pereira et al., 2007).

After the detection of Morris water maze test, the rats were divided for the Western Blotting and immunohistochemistry experiments.

### Reactive oxygen species assay

The oxidant-sensing fluorescent probe 2’,7’-dichlorodihydrofluorescein diacetate (DCFH-DA) is a non-polar dye that is converted to highly fluorescent 2’,7’-dichlorodihydrofluorescein (DCF) when oxidized by intracellular ROS (Rastogi et al., 2010). ROS levels were assessed by measuring DCF levels (Zhang M. et al., 2020).

As previously described (Zhang M. et al., 2020), DCF levels were evaluated at 24 h, 3 days, and 21 days (*n* = 5/group per time point) during PTZ-induced epileptogenesis. After administering pentobarbital sodium (50 mg/kg, intraperitoneally, CAS, 57-33-0, Xiya Reagent, Chengdu, China), the brain was quickly removed, and the cortex and hippocampus were separated on ice, following which 0.01M PBS (10 μL/mg) was added to the samples, which were then cut using scissors on ice. Sequentially, the cut issue was filtered through a stainless steel mesh (200 mesh/inch) for the single-cell suspensions (Cheng et al., 2021). In accordance with the instructions of the ROS Assay Kit (Beyotime, S0033, Shanghai, China), 500 μL DCFH-DA diluted with serum-free culture medium (1:1000, 10 μM/L) were added to the single cell suspensions (250 μL) for 40 min incubation at 37°C without daylight. After washing three times with 0.01M PBS, the fluorescence intensity of 200 μL samples was analyzed using a fluorescence microplate reader (Synery H1; Thermo, Waltham, MA, USA) at 488 nm (excitation wavelength) and 525 nm (emission wavelength).

### Determination of mitochondrial reactive oxygen species

Similar to the ROS analysis, mito-SOX was detected at 24 h, 3 days, and 21 days (*n* = 5/group per time point) during epileptogenesis. After single-cell suspensions were prepared, 250 μL suspensions were added to 1 mL mito-SOX (M36008, 5 μM, Thermo Fisher, Waltham, MA, USA) and incubated for 10 min without light at 37°C. After three washing with 0.01M PBS, the fluorescence intensity was measured using a fluorescence microplate reader and a flow cytometer (BD FACSCantoTM; BD Biosciences, Piscataway, NJ, USA) at 510 nm (excitation wavelength) and 580 nm (emission wavelength).

### Iron content detection

As previously described (Zhang M. et al., 2020), the cortex and hippocampus of each rat were isolated at 24 h, 3 days, and 21 days (*n* = 5/group per time point) during epileptogenesis. Subsequently, the iron content in the tissues was measured using an iron content detection kit (DIFE008, Bioassay Systems, NC, USA).

### Fluoro-jade B staining

FJB staining was used to assess neuronal damage (Anderson et al., 2005). As previously described (Zhang M. et al., 2020), rats from each group were cardiac-perfused, and tissue slices were prepared at 24 h, 3 days, and 21 days (*n* = 5/group per time point) during epileptogenesis. The slices were stained using an FJB staining kit (AG310, Millipore, NC, USA). Positive signals of FJB were obtained through fluorescence microscopy (Olympus, Tokyo, Japan) and were manually counted.

### Western blot analysis

As previously described (Zhang M. et al., 2020), the cortex and hippocampus of each rat were isolated on days 3 and 21 during epileptogenesis. Proteins were extracted using a reagent kit (P0012, Beyotime, China). Rabbit monoclonal antibodies against ferroportin 1 (FPN1, 1:2000, ab58695, Abcam, Cambridge, UK), iron regulatory protein 1 (IRP1, 1:1000, ab236773, Abcam, UK), microtubule-associated protein light chain 3beta (LC3B, 1:2000; ab48394, Abcam, UK), caspase-3 (1:1000, 9662, Cell Signaling Technology, Boston, MA, USA), activated caspase-3 (1:1000, ab2302, Abcam, UK), and glyceraldehyde 3-phosphate dehydrogenase (GAPDH, 1:3000, AB-P-R001, Kangcheng, Nanjing, China) were used for western blot analysis. Strip images were acquired using an image analyzer (ImageQuant LAS 500, GE Healthcare, Uppsala, Sweden). Grayscale analysis of target strips was performed using ImageJ V.1.37 software (National Institutes of Health, Bethesda, MD, USA).

### Immunohistochemistry

As previously described (Zhang M. et al., 2020), tissue slices were obtained at 24 h, 3 days, and 21 days, and treated with IRP1/DMT1/4’,6-diamidino-2-phenylindole (DAPI) and micro-tubule-associated protein light chain 3B (LC3B)/translocase of outer mitochondrial membrane 20 (TOMM20)/DAPI immunohistochemical staining. Rabbit monoclonal antibodies against IRP1 (1:200, ab236773, Abcam, UK) and LC3B (1:200, ab48394, Abcam, UK) and mouse monoclonal antibodies against TOMM20 (1:200, ab56783, Abcam, UK) and DMT1 (1:200, ab55735, Abcam, UK) were used for immunohistochemistry. Images were obtained using a fluorescence microscope (Olympus, Japan) and analyzed using ImageJ V.1.37 software (National Institutes of Health, USA).

### Statistical analyses

Before the experiments, the number of rats in each group was estimated using a balanced one-way analysis of variance (ANOVA). All values are expressed as mean ± standard error of the mean (SEM), and statistical analysis of the data was performed using SPSS (Version 25.0; SPSS Inc., Chicago, IL, USA). The number and cumulative duration of seizures, and latency to reach the platform in the Morris water maze were analyzed using two-way RM-ANOVA. The difference in single variables between the two groups was analyzed by unpaired *t*-test, and the difference in single variables in multiple groups was analyzed using one-way ANOVA. In all analyses, statistical significance was set at *P* < 0.05.

## Results

### Pentylenetetrazole-induced epileptogenesis and cognitive deficits

**FIGURE 1:**
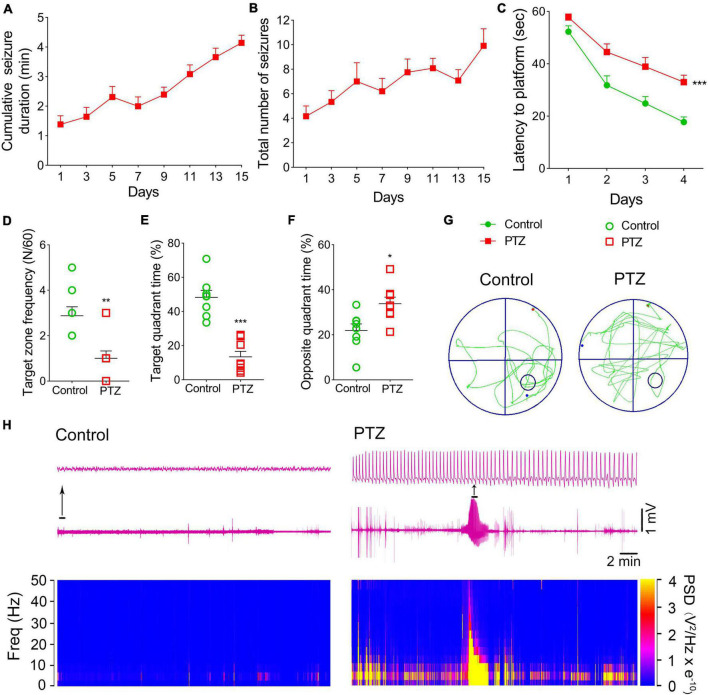
Pentylenetetrazole (PTZ)-induced epileptogenesis and cognitive deficits. **(A)** Cumulative seizure duration. **(B)** Total number of seizures. **(C)** Latency to the platform (two-way RM-ANOVA). **(D)** Frequency of platform crossings. **(E)** Target quadrant time (%). **(F)** Opposite quadrant time (%). **(G)** Representative platform exploration trajectories. **(H)** Representative EEGs and power spectrum density. Data are presented as mean ± SEM. *P < 0.05, **P < 0.01, and ***P < 0.001, compared with controls (unpaired *T*-tests).

Pentylenetetrazole (PTZ)-induced epileptogenesis and cognitive deficits were assessed in SD rats. In the PTZ group (*n* = 12), seizures (cumulative seizure duration, Figure 1A; total number of seizures, Figure 1B) were observed during epileptogenesis. The representative EEGs and analysis of the frequency spectrum and power spectrum density are shown in Figure 1H.

The Morris water maze experiment was performed on Day 16 to assess the learning and memory abilities of rats in PTZ-induced epileptogenesis. Compared to the control group (*n* = 8), the PTZ group (*n* = 8) had a longer latency to reach the platform (*P* < 0.001, Figure 1C), crossed the platform less frequently (*P* = 0.003, Figure 1D), spent lesser time in the target quadrant (*P* < 0.001, Figure 1E), and spent more time in the opposite quadrant (*P* = 0.012, Figure 1F). These results indicated that PTZ treatment leads to defects in learning and memory. The representative platform exploration trajectories for each group are shown in Figure 1G.

### Iron levels significantly increased due to pentylenetetrazole treatment

**FIGURE 2:**
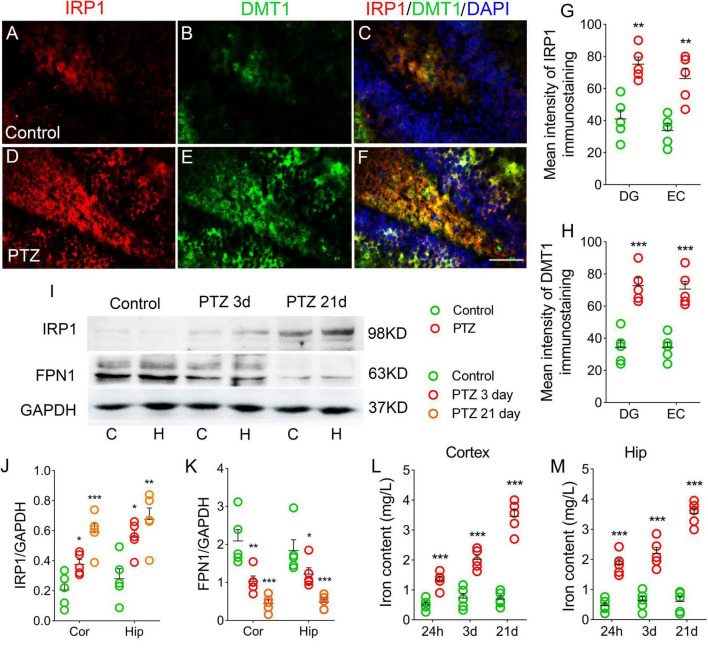
Pentylenetetrazole (PTZ) significantly increased iron levels. **(A–F)** Increased fluorescence intensity of IRP1 (red) and DMT1 (green) in the dentate gyrus (DG) region during PTZ-induced epileptogenesis (blue, DAPI). Bar = 80 μm. **(G,H)** Quantified changes in IRP1 and DMT1 (*n* = 5/group). **(I)** Expression of IRP1 and FPN1 in the hippocampus and cortex estimated by western blotting (*n* = 5/group). **(J,K)** Normalized intensity of IRP1 and FPN1 relative to GAPDH (one-way ANOVA). **(L,M)** Levels of iron (*n* = 5/timepoint). **P < 0.01 and ***P < 0.001, compared with controls (unpaired *T*-tests).

The iron content and expression of iron-related proteins were detected in each group. The immunohistochemical results showed that the immunofluorescence intensity of hippocampus and cortex IRP1 was significantly higher in the PTZ-treated group than in the control group [e.g., dentate gyrus (DG), *P* = 0.001, Figures 2A,C,D,F,G, Supplementary Figures 1A–F]. The DMTI levels were also observed to increase (*P* < 0.001, Figures 2B,E,H). Western blotting results further confirmed that the levels of IRP1 were significantly higher in the PTZ group than in the control group (3 days, cortex, *P* = 0.025; hippocampus, *P* = 0.011; 21 days, cortex, *P* < 0.001; hippocampus, *P* = 0.001; Figures 2I,J). However, the expression of FPN1, which is responsible for iron outflow (Minor et al., 2019), was lower in the PTZ group than in the control group (Figures 2I,K). We also examined the iron content in the cortex and hippocampus and found that iron levels were significantly higher in the PTZ group than in the control group (24 h, 3 days, and 21 days, *P* < 0.001, Figures 2L,M).

### Increased 2’,7’-dichlorodihydrofluorescein and mito-SOX levels during pentylenetetrazole-induced epileptogenesis

**FIGURE 3:**
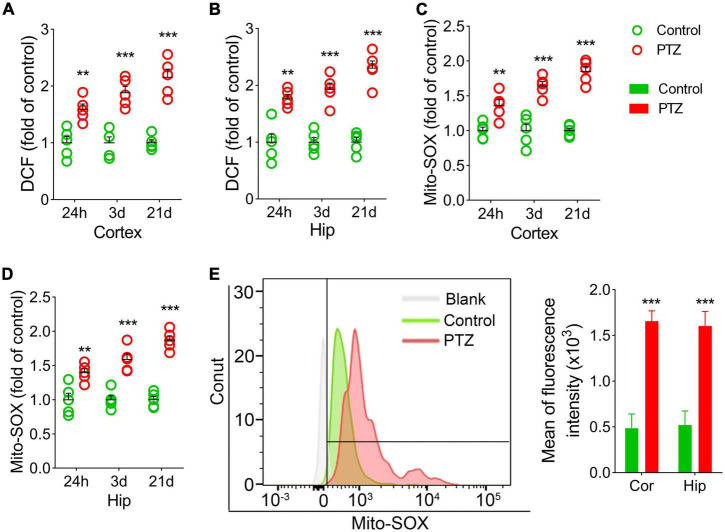
Increased 2’,7’-dichlorodihydrofluorescein (DCF) and mito-SOX levels during pentylenetetrazole (PTZ)-induced epileptogenesis. Increased DCF **(A,B)** and mito-SOX **(C,D)** levels in the hippocampus and cortex during epileptogenesis (*n* = 5/group). **(E)** Flow cytometry-based quantification of mito-SOX levels. **P < 0.01 and ***P < 0.001, compared with controls (unpaired *T*-tests).

We also examined the DCF and mito-SOX levels in each group. The results showed that cortex and hippocampus DCF levels were significantly higher in the PTZ group than in the control group (24 h, cortex, *P* = 0.004; hippocampus, *P* = 0.001; 3 days, *P* < 0.001; 21 days, *P* < 0.001; Figures 3A,B). Similar increases in mito-SOX levels were also observed (cortex, *P* < 0.001; hippocampus, *P* < 0.001; Figures 3C,D). Representative mito-SOX flow cytometry results are shown in Figure 3E.

### Increased autophagy/mitophagy and neuronal injury during pentylenetetrazole-induced epileptogenesis

LC3B is a marker of autophagosomes, which can initiate autophagy (Vives-Bauza and Przedborski, 2011). LC3B levels are clearly correlated with mitophagy (Nakatogawa et al., 2007; Weidberg et al., 2011; Klionsky et al., 2012). TOMM20 is a receptor on the outer mitochondrial membrane (Wu et al., 2018). Co-labeling of LC3B and TOMM20 indicates mitophagy (Wu et al., 2018). Counterstaining of LC3B and TOMM20 was performed to evaluate the mitophagy.

**FIGURE 4:**
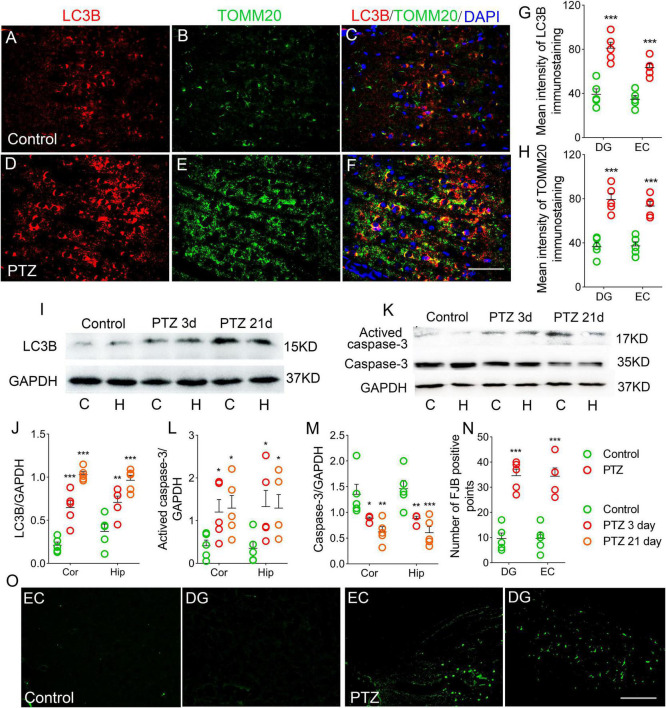
Increased autophagy/mitophagy and neuronal injury in pentylenetetrazole (PTZ)-induced epileptogenesis. **(A–F)** Increased fluorescence intensity of LC3B (red) and TOMM20 (green) in the EC region during epileptogenesis (*n* = 5/group; blue, DAPI). Bar = 60 μm. **(G,H)** Quantified changes in LC3B and TOMM20 (*n* = 5/group). **(I)** Expression of LC3B in the hippocampus and cortex estimated by western blotting (*n* = 5/group). **(J)** Normalized intensity of LC3B relative to GAPDH. **(K–M)** Levels of caspase-3 and activated caspase-3 in the hippocampus and cortex estimated by western blotting (*n* = 5/group). **(N,O)** Positive FJB signals counted in each group. *P < 0.05 and ***P < 0.001, all compared with controls (panels **J–M**, one-way ANOVA; the others, unpaired *T*-tests].

During epileptogenesis, we found a significant increase in the levels of LC3B and TOMM20 in the hippocampus and cortex (*P* < 0.001), and the majority of LC3B was co-labeled with TOMM20 (Figures 4A–H; Supplementary Figures 1M–R). Similar increases in LC3B levels were confirmed by western blotting (*P* < 0.001; Figures 4I,J).

In addition, in the PTZ group, the levels of activated caspase-3 were significantly higher (Figures 4K,L), and caspase-3 levels were decreased (Figures 4K,M) in the cortex and hippocampus compared with the control group. Neuronal damage was assessed using FJB staining (Anderson et al., 2005), which confirmed that FJB-positive signals were significantly increased by PTZ treatment (*P* < 0.001, Figures 4N,O).

### Xenon treatment reduced severity of pentylenetetrazole-induced epileptic development and cognitive deficits

**FIGURE 5:**
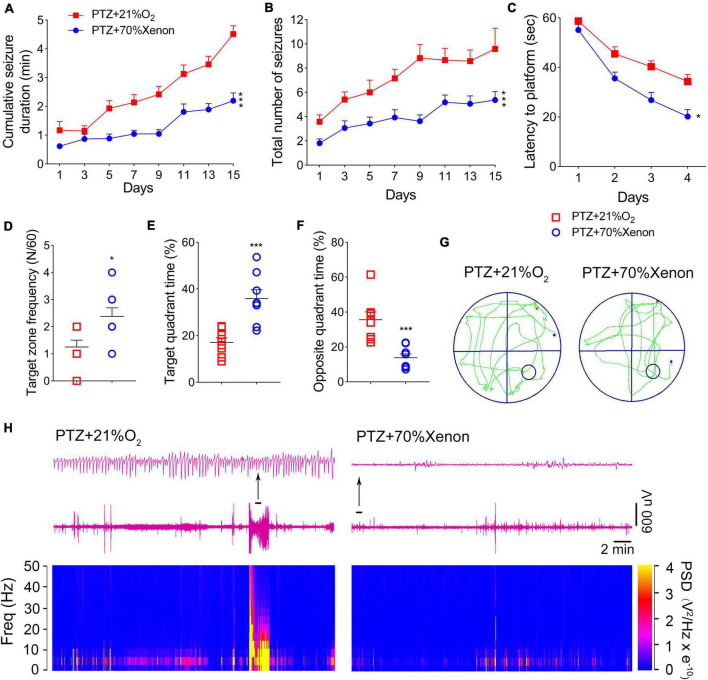
Xenon treatment reduced severity of pentylenetetrazole (PTZ)-induced epileptogenesis and cognitive deficits. **(A)** Cumulative seizure duration. **(B)** Total number of seizures. **(C)** Latency to the platform. **(D)** Frequency of platform crossings. **(E)** Target quadrant time (%). **(F)** Opposite quadrant time (%). **(G)** Representative tracking. **(H)** Representative electroencephalography (EEGs) and power spectrum density. Data are presented as mean ± SEM. *P < 0.05 and ***P < 0.001, compared with controls (panels **A–C**, two-way RM-ANOVA; panels **D–F**, unpaired *T*-tests).

Rats in the xenon group (*n* = 16) received xenon treatment for 30 min immediately after PTZ injection, whereas the control group (*n* = 12) received 21% O_2_/79% N_2_ treatment. A comparison of seizure frequency and duration between PTZ and PTZ + 21% O_2_ groups showed no significant difference (cumulative seizure duration, *P* = 0.841; total number of seizures, *P* = 0.824, Supplementary Figures 2A,B). The results showed that cumulative seizure duration (*P* < 0.001, Figure 5A) and total number of seizures (*P* < 0.001, Figure 5B) were significantly lower in the xenon group than in the control group. EEG analysis suggested a similar attenuation by xenon treatment (Supplementary Figures 3A,B).

The Morris water maze results indicated that xenon treatment attenuated learning and memory impairments induced by PTZ treatment. The xenon group showed shorter latency to reach the platform (*P* = 0.016, Figure 5C), crossed the platform more frequently (*P* = 0.016, Figure 5D), spent more time in the target quadrant (*P* < 0.001, Figure 5E), and spent less time in the opposite quadrant (*P* < 0.001, Figure 5F). While the difference between the naïve and xenon-treated rats indicated that xenon partly, but not completely rescued the cognition impairment induced by PTZ kindling (Supplementary Figure 4). The representative EEGs, analysis of the frequency spectrum and power spectrum density, and platform exploration trajectory of each group are shown in Figures 5G,H.

### Xenon treatment reduced the iron accumulation caused by pentylenetetrazole treatment

**FIGURE 6:**
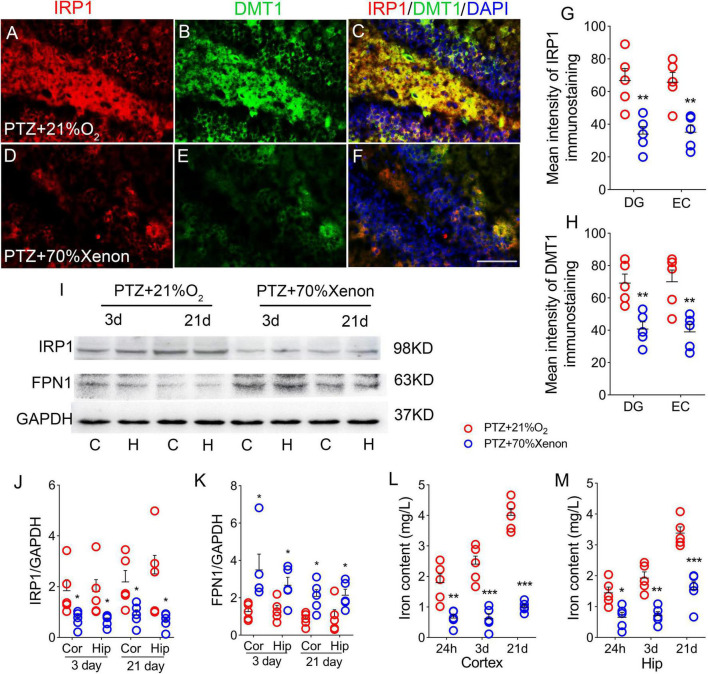
Xenon treatment reduced the iron accumulation caused by pentylenetetrazole (PTZ). **(A–F)** Increased levels of IRP1 (red) and DMT1 (green) in the dentate gyrus (DG) region caused by PTZ were attenuated by xenon treatment (blue, DAPI). Bar = 80 μm. **(G,H)** Quantified changes in IRP1 and DMT1 (*n* = 5/group). **(I)** Expression of IRP1 and FPN1 in the hippocampus and the cortex estimated by western blotting (*n* = 5/group). **(J,K)** Normalized intensity of IRP1 and FPN1 relative to GAPDH (one-way ANOVA). **(L,M)** Levels of iron (*n* = 5/timepoint). *P < 0.05, **P < 0.01, and ***P < 0.001, compared with controls (unpaired *T*-tests).

The results of immunohistochemistry showed that the immunofluorescence intensity of IRP1 was significantly lower in the hippocampus and cortex (e.g., DG, *P* = 0.005; Figures 6A,C,D,F,G; EC, *P* = 0.003; Figure 6G) due to the xenon mixture inhalation. Similarly, decreased levels of DMT1 were observed (DG, Figures 6B,E,H, Supplementary Figures 1G–L; EC, Figure 6H) in xenon-treated rats. Western blotting results verified the decrease in IRP1 levels (3 days, cortex, *P* = 0.038; hippocampus, *P* = 0.046; 21 days, cortex, *P* = 0.042; hippocampus, *P* = 0.038; Figures 6I,J), while FPN1 levels were higher in the xenon group than in the control group (Figures 6I,K). The iron content results indicate that xenon treatment reversed PTZ-induced iron accumulation (24 h, cortex, *P* = 0.005; hippocampus, *P* = 0.012; 3 days, cortex, *P* < 0.001; hippocampus, *P* = 0.001; 21 days, *P* < 0.001; Figures 6L,M).

### Xenon treatment reduced the elevated levels of 2’,7’-dichlorodihydrofluorescein and mito-SOX caused by pentylenetetrazole administration

**FIGURE 7:**
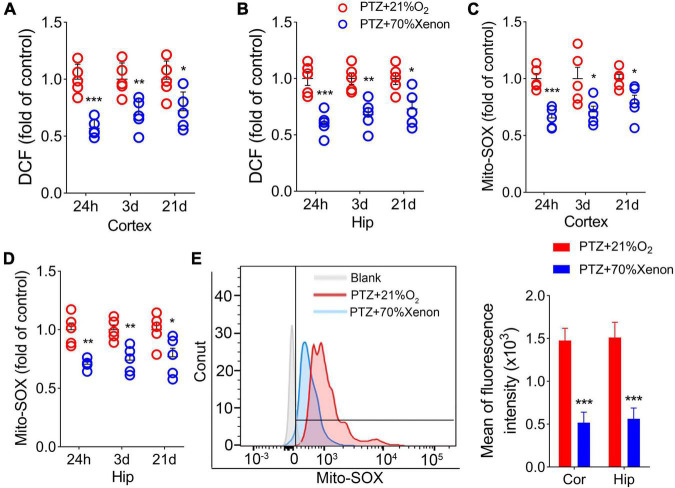
Xenon treatment reduced the elevated levels of 2’,7’-dichlorodihydrofluorescein (DCF) and mito-SOX caused by pentylenetetrazole (PTZ) administration. Decreased levels of DCF **(A,B)** and mito-SOX **(C,D)** in the hippocampus and cortex due to xenon treatment (*n* = 5/group). **(E)** Flow cytometry-based quantification of hippocampal mito-SOX levels. *P < 0.05, **P < 0.01, and ***P < 0.001, compared with controls (unpaired *T*-tests).

The DCF results showed a significant reduction in the cortex (Figure 7A) and hippocampus (Figure 7B) in the xenon group compared with the control group. A similar decrease in mito-SOX levels was observed after xenon treatment (Figures 7C,D). Representative mito-SOX flow cytometry results are shown in Figure 7E.

### Xenon treatment prevented pentylenetetrazole-induced autophagy/mitophagy and neuronal injury

**FIGURE 8:**
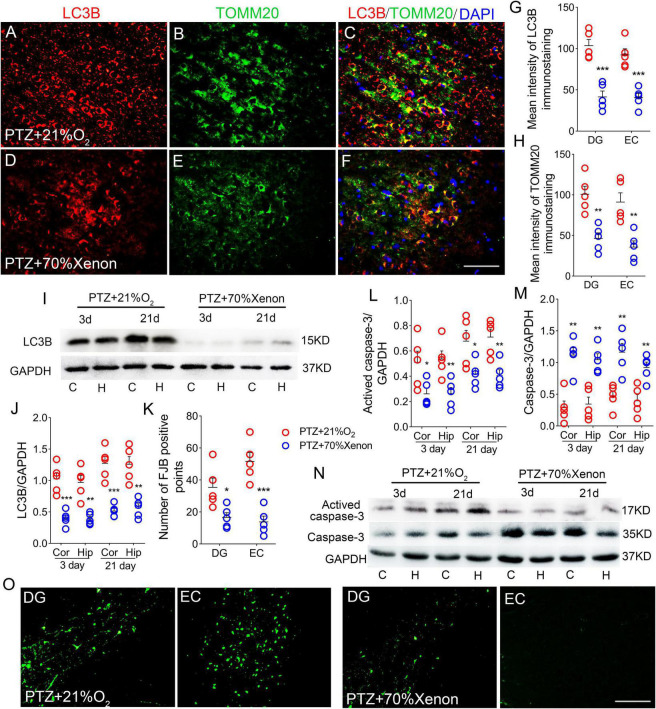
Xenon treatment prevented pentylenetetrazole (PTZ)-induced autophagy/mitophagy and neuronal injury. **(A–F)** Decreased fluorescence intensity of LC3B (red) and TOMM20 (green) in the EC region due to xenon treatment (*n* = 5/group; blue, DAPI). Bar = 60 μm. **(G,H)** Quantified changes in LC3B and TOMM20 (*n* = 5/group). **(I)** Expression of LC3B in the hippocampus and cortex estimated by western blotting (*n* = 5/group). **(J)** Normalized levels of LC3B relative to GAPDH. **(L–N)** Levels of caspase-3 and activated caspase-3 in the hippocampus and cortex estimated by western blotting (*n* = 5/group). **(K,O)** Positive FJB signals counted in each group. *P < 0.05, **P < 0.01, and ***P < 0.001, compared with controls (unpaired *T*-tests).

Immunohistochemical results showed that xenon treatment led to reduced levels of LC3B (DG, *P* < 0.001; EC, *P* < 0.001; Figures 8A,C,D,F,G; Supplementary Figures 1S–X) and TOMM20 (DG, *P* = 0.002; EC, *P* = 0.004; Figures 8B,E,H; Supplementary Figures 1S–X). Western blotting results further confirmed the significant a decrease in LC3B expression (3 days, cortex, *P* < 0.001; hippocampus, *P* = 0.001; 21 days, cortex, *P* < 0.005; hippocampus, *P* = 0.001; Figures 8I,J) after xenon treatment. Moreover, in the xenon group, the levels of activated caspase-3 were significantly lower and caspase-3 levels were higher than those in the control group at Days 3 and 21 (Figures 8L–N). Meanwhile, FJB-positive signals were significantly decreased (DG, *P* = 0.033; EC, *P* < 0.001; Figures 8K,O) in the xenon group compared with those in the control group.

## Discussion

Our results demonstrate a strong link between oxidative stress, iron accumulation, and neuronal damage during PTZ-induced epileptogenesis. However, immediate xenon treatment following each PTZ injection significantly reduced PTZ-induced oxidative and iron stress, accompanied by neuronal damage, seizures, and cognitive dysfunction.

Epilepsy is a common nervous system disease. However, clinical therapy for epilepsy has limitations, and curative effects and adverse reactions tend to be unsatisfactory (Berg et al., 1996; Schmidt and Loscher, 2005). Moreover, partial cases of epilepsy may develop into refractory epilepsy (Perucca and Gilliam, 2012; Engel, 2017). Therefore, an effective and safe therapeutic strategy for preventing epileptic development is important.

Pentylenetetrazole (PTZ) is a chloride channel antagonist of GABA-A receptor, which leads to injuries in the hippocampus and is widely used for kindling epileptic models (Ahmadi et al., 2017). During PTZ-kindling, seizures are induced by overexcitation (Schröder et al., 1993).

Elevated levels of glutamate-induced over-excitation and oxidative stress are vital characteristics of epileptic development (During and Spencer, 1993). During this progression, the overexcitation of NMDA receptors upregulates the expression of DMT1 (Wang et al., 2021), a protein that regulates iron influx, thereby increasing the level of cellular iron (Cesar et al., 2012). In addition, excess ROS further exacerbates iron accumulation by upregulating IRP1, which maintains intracellular iron homeostasis (Theil and Eisenstein, 2000), and further downregulates FPN1 (which is responsible for iron efflux) and upregulates DMT1 (which is responsible for iron influx) (Cesar et al., 2012). The iron content in the brain is regulated by iron-regulated proteins IRP1, DMT1, and FPN1 (Zhang et al., 2009; Song et al., 2010; Jiang et al., 2017). Eventually, iron accumulation-induced iron stress occurs following overexcitation and oxidative stress.

Iron metabolism in the brain is closely related to brain development and function. Past studies have confirmed a close relationship between iron levels and epilepsy (Cai and Yang, 2021; Zimmer et al., 2021). Intracortical injections of Fe^3+^ can induce seizures (Das et al., 2017). High levels of iron generate several highly reactive hydroxyl radicals through the Fenton reaction (Nishizaki and Iwahashi, 2015), has a disruptive effect on lipids and proteins, and can trigger epilepsy and neuronal apoptosis by activating the calpain and caspase-3 pathways (Baudry and Bi, 2016). Obviously, there is a mutual promotion between iron accumulation and oxidative stress (Molinari et al., 2019).

Mitochondria are vital sites for ROS production, and approximately 90% of ROS are produced by mitochondria (Brand, 2010; Yan et al., 2013). In contrast, mitochondria are sensitive and vulnerable to ROS (Shimura, 2021) and produce antioxidant enzymes to clear excess ROS and maintain redox homeostasis. However, once this balance is disrupted and mitochondria are damaged, large amounts of ROS are produced, and triggering mitophagy (Sasaki et al., 2019). Mitochondrial dysfunction and mitophagy are of the main features of epilepsy (Chang and Yu, 2010). Immoderate mitophagy can change transient Ca^2+^ states and promote excess production of ROS (Han et al., 2011; Zhang Y. et al., 2020), ultimately triggering apoptosis by activating the calpain and caspase-3 pathways and aggravating neuronal injury (Baudry and Bi, 2016). In temporal lobe epilepsy, mitochondrial dysfunction-induced oxidative stress leads to increased neuronal excitation and promotes epileptogenesis (Rowley and Patel, 2013). Previous studies have suggested a possible contribution of oxidative stress, iron stress, and related apoptosis, neuronal injury, and mitophagy during epileptogenesis. Our results confirmed this hypothesis regarding PTZ-induced epileptic development.

According to our previous review (Zhang et al., 2021), xenon exerts neuroprotective effects involving not only the inhibition of *N*-methyl-daspartic acid (NMDA) receptors, attenuation of excitotoxicity, and NMDA receptor-related effects (Wilhelm et al., 2002), such as antioxidative effects, reduced activation of microglia, and Ca^2+^-dependent mechanisms, as well as interaction with certain ion channels. Previous reports have confirmed the significant neuroprotective effects of xenon through antioxidant action (Yang et al., 2016). Xenon not only restores oxidative stress to basic levels, but also reduces mitochondrial damage mediated by oxidative stress through direct and indirect effects (Lavaur et al., 2017). Our results confirmed that xenon treatment could reduce the elevated levels of oxidative stress and iron, and the related apoptosis, neuronal injury, and mitophagy were attenuated, accompanied by relieved seizures and cognitive impairment in PTZ-kindling rats. These experiment results indicate that the reduction in closely related oxidative stress and iron stress may be vital contributors to protection underlying xenon treatment (Supplementary Figure 5).

Caspases are a family of proteins associated with inflammation and apoptosis (D’Amelio et al., 2010). Caspase-3 is considered a key member of the family that mediates the neuronal apoptosis pathway, and activated caspase-3 is thought to be an important indicator of neuronal apoptosis (D’Amelio et al., 2010; Zhao et al., 2021). According to the previous study, activation of caspase-3 was generally induced by oxidative stress injury (Kim et al., 2020). It has been reported that excessive production of ROS causes neuronal injury and then activates caspase-3 mediated apoptosis signaling pathways (Zhao et al., 2021). Our results showed that xenon treatment alleviated cell apoptosis, which may be related to the reduction in oxidative stress in PTZ-induced epileptogenesis.

The hippocampus and subregions of cortex are key brain regions that are closely related to epileptic development, and learning and memory (Lado et al., 2002; Coutureau and Scala, 2009; Vismer et al., 2015; Hainmueller and Bartos, 2020). It has been verified that EC is responsible for receiving information and transmitting it to the DG, which is believed to be a vital region related to cognitive function (Hainmueller and Bartos, 2020). In our experiments, we found significant damage to these regions after successive PTZ injections, accompanied by seizures and cognitive defects. However, after xenon treatment, damage to the DG and EC was alleviated, seizures were attenuated, and cognitive functions were improved. The results indicated that reduced neuronal damage may contribute to anti-seizure effects and improvements of cognitive function caused by xenon (Supplementary Figure 5).

However, exposing epileptic patients to xenon-rich environments every time they develop an episode is impractical. Therefore, after determining the protective effects of xenon, the roles of xenon in more clinically relevant paradigms, for example, different time delays, or interictal treatment, are expected.

In summary, our study confirmed the significant anti-seizure and neuroprotective effects of xenon on PTZ-induced epileptogenesis. Furthermore, reducing iron stress and oxidative stress may be the potential mechanisms underlying xenon protection. Our study advances the application of xenon in prevention of epileptogenesis.

## Data availability statement

The original contributions presented in this study are included in the article/Supplementary material, further inquiries can be directed to the corresponding authors.

## Ethics statement

This animal study was reviewed and approved by Binzhou Medical University Animal Experimentation Committee (approval no. 2020002).

## Author contributions

MZ and YC: study design, data acquisition, and analysis. HS and SL: study conception, design, data interpretation, and manuscript drafting. YZ, YY, HH, XM, and XF: PTZ-induced epileptogenesis model preparation and data acquisition. All authors contributed to the article and approved the submitted version.

## Supplementary material

Supplementary Figure 1 The immunofluorescence in the dentate gyrus (DG) and EC (20× magnification images). **(A–L)** Fluorescence intensity of IRP1 (red) and DMT1 (green) in the DG region during pentylenetetrazole (PTZ)-induced epileptogenesis (blue, DAPI). Bar = 30 μm. **(M–X)** Fluorescence intensity of LC3B (red) and TOMM20 (green) in the EC region during epileptogenesis (blue, DAPI). Bar = 30 μm.

Supplementary Figure 2 Comparison of seizure frequency and duration between pentylenetetrazole (PTZ) group and PTZ + 21% O_2_ group. **(A)** Cumulative seizure duration and **(B)** total number of seizures in PTZ-treated group (two-way RM-ANOVA).

Supplementary Figure 3 The analysis of electroencephalography (EEGs) in rats treated with and without xenon. **(A)** Cumulative seizure duration, and **(B)** total number of seizures analyzed by EEGs (two-way RM-ANOVA). ***P* < 0.01, and ****P* < 0.001.

Supplementary Figure 4 Xenon treatment partially alleviated the cognition impairment induced by pentylenetetrazole (PTZ) kindling. **(A)** Latency to the platform (two-way RM-ANOVA). **(B)** Frequency of platform crossings. **(C)** Target quadrant time (%). **(D)** Opposite quadrant time (%). **P* < 0.05, compared with controls (unpaired *T*-tests).

Supplementary Figure 5 The roles and mechanisms of xenon in pentylenetetrazole (PTZ)-induced seizures and neuronal damage.
