# Supplementary figures and images for: Attenuated iron stress and oxidative stress may participate in anti-seizure and neuroprotective roles of xenon in pentylenetetrazole-induced epileptogenesis
Source: Front Cell Neurosci. 2022 Nov 17;16:1007458. doi: 10.3389/fncel.2022.1007458 (PMC9712733; doi:10.3389/fncel.2022.1007458)

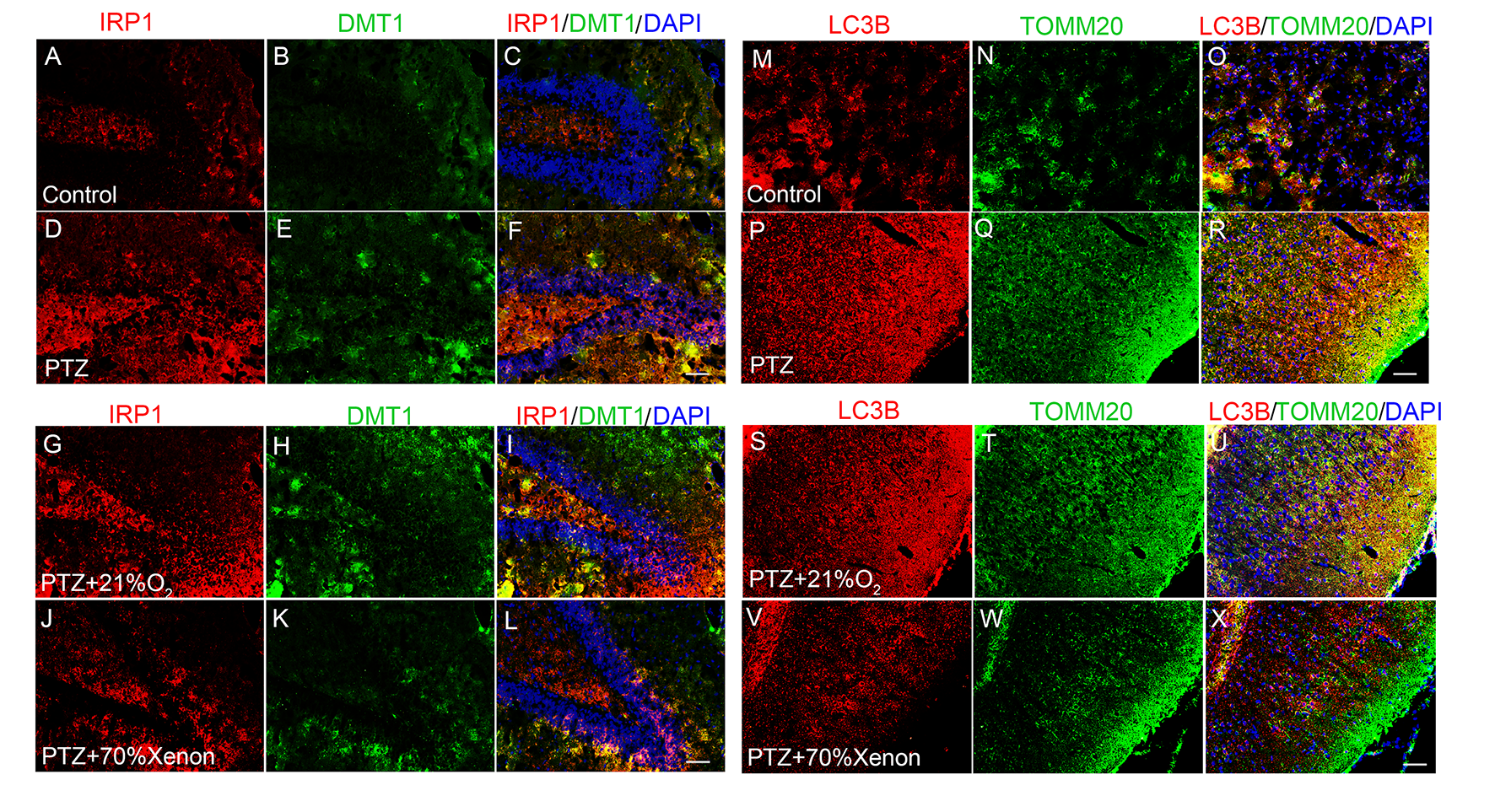

Supplement: Supplementary Figure 1 — The immunofluorescence in the dentate gyrus (DG) and EC (20× magnification images). (A–L) Fluorescence intensity of IRP1 (red) and DMT1 (green) in the DG region during pentylenetetrazole (PTZ)-induced epileptogenesis (blue, DAPI). Bar = 30 μm. (M–X) Fluorescence intensity of LC3B (red) and TOMM20 (green) in the EC region during epileptogenesis (blue, DAPI). Bar = 30 μm. [file Image_1.TIF]

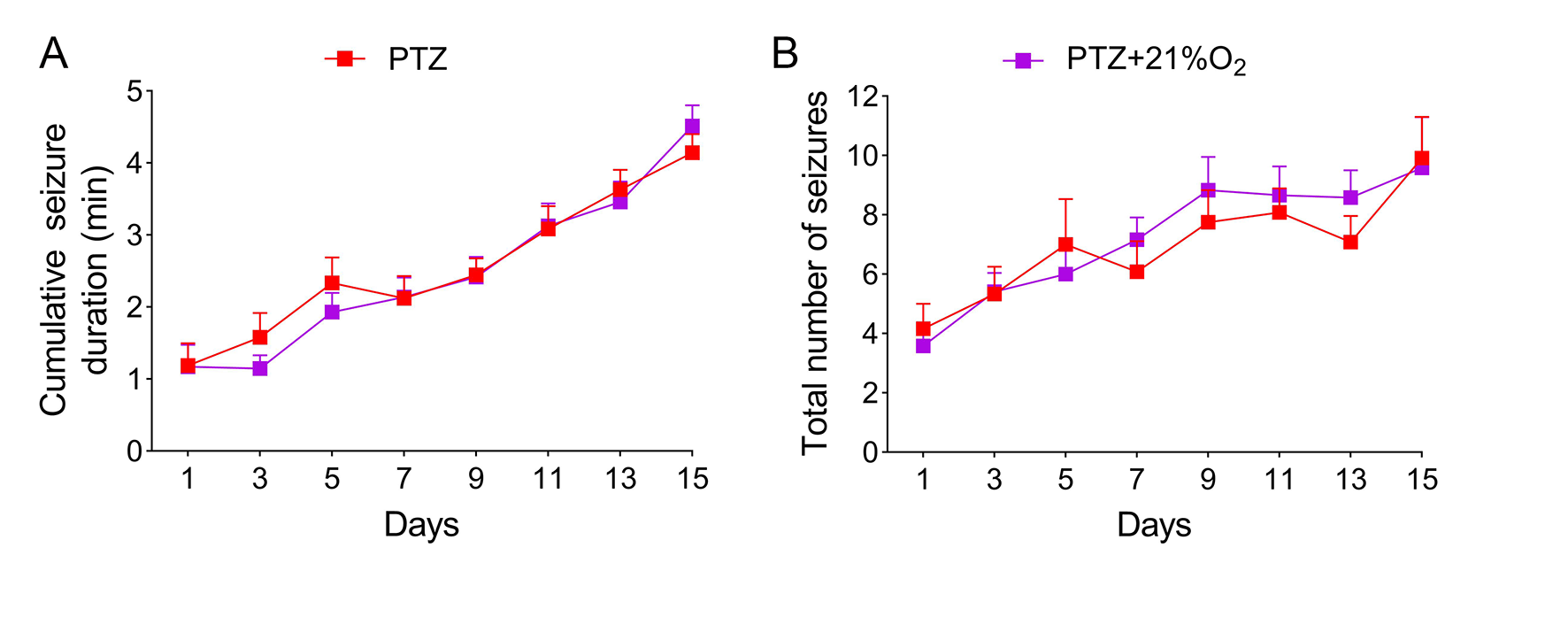

Supplement: Supplementary Figure 2 — Comparison of seizure frequency and duration between pentylenetetrazole (PTZ) group and PTZ + 21% O2 group. (A) Cumulative seizure duration and (B) total number of seizures in PTZ-treated group (two-way RM-ANOVA). [file Image_2.TIF]

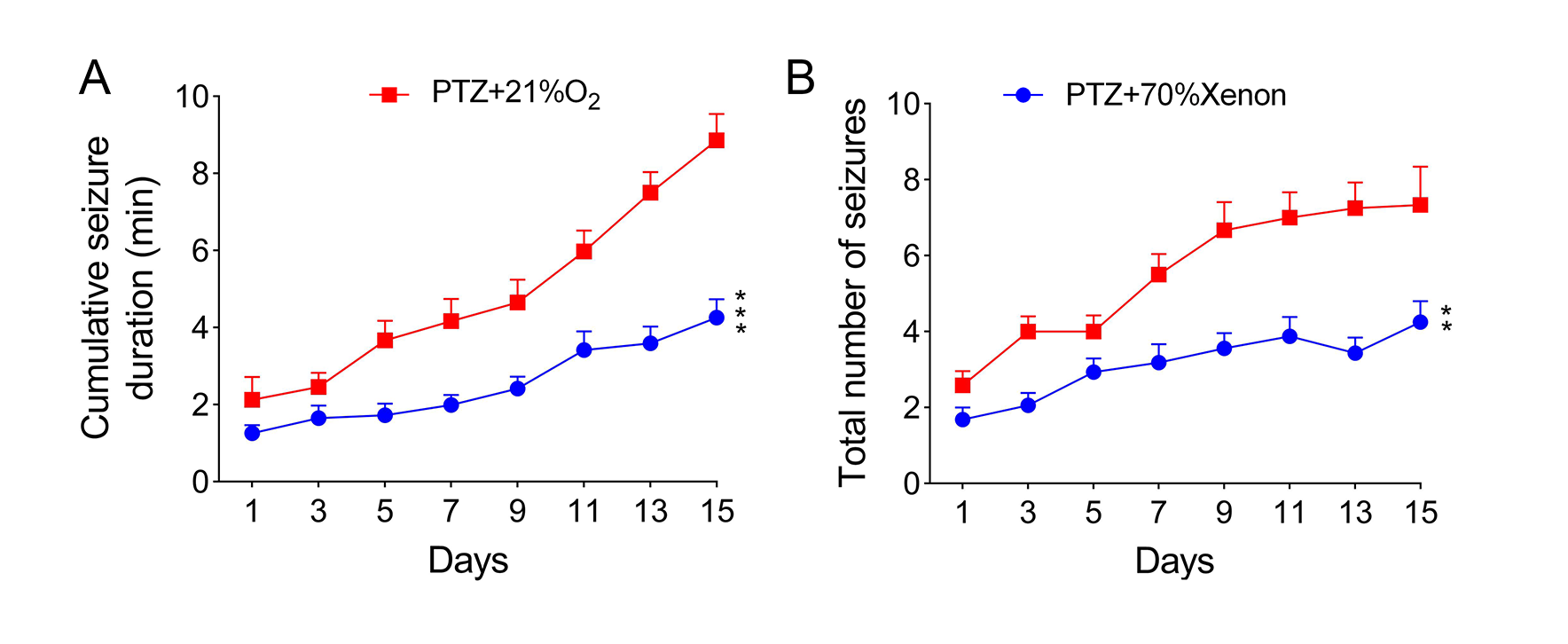

Supplement: Supplementary Figure 3 — The analysis of electroencephalography (EEGs) in rats treated with and without xenon. (A) Cumulative seizure duration, and (B) total number of seizures analyzed by EEGs (two-way RM-ANOVA). **P < 0.01, and ***P < 0.001. [file Image_3.TIF]

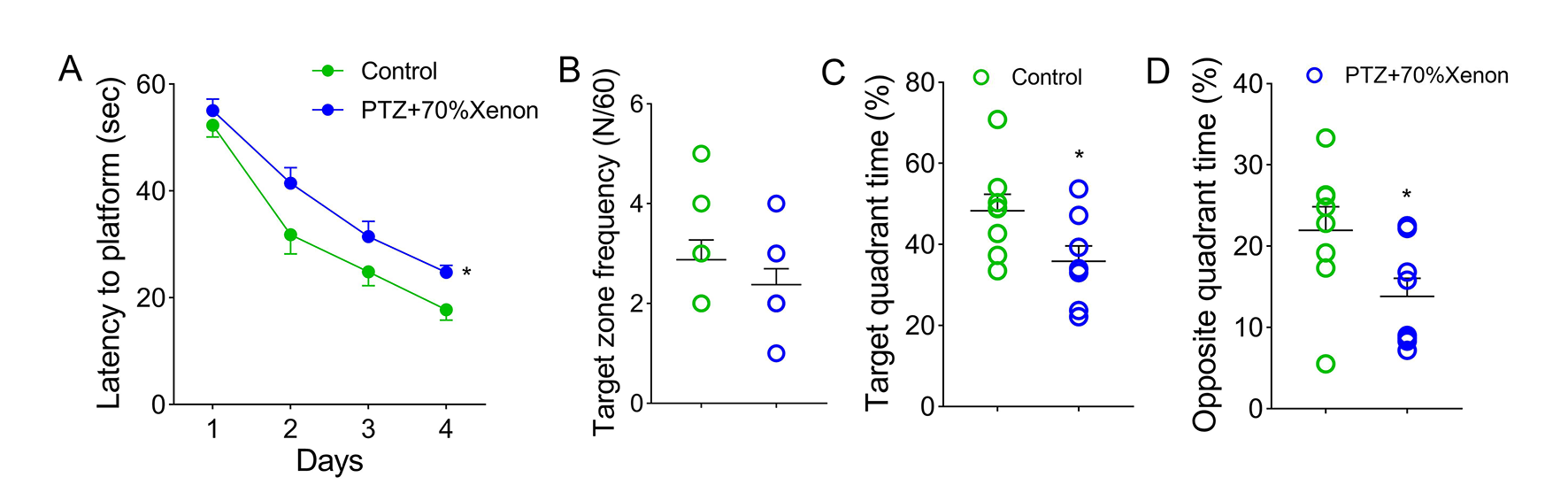

Supplement: Supplementary Figure 4 — Xenon treatment partially alleviated the cognition impairment induced by pentylenetetrazole (PTZ) kindling. (A) Latency to the platform (two-way RM-ANOVA). (B) Frequency of platform crossings. (C) Target quadrant time (%). (D) Opposite quadrant time (%). *P < 0.05, compared with controls (unpaired T-tests). [file Image_4.TIF]

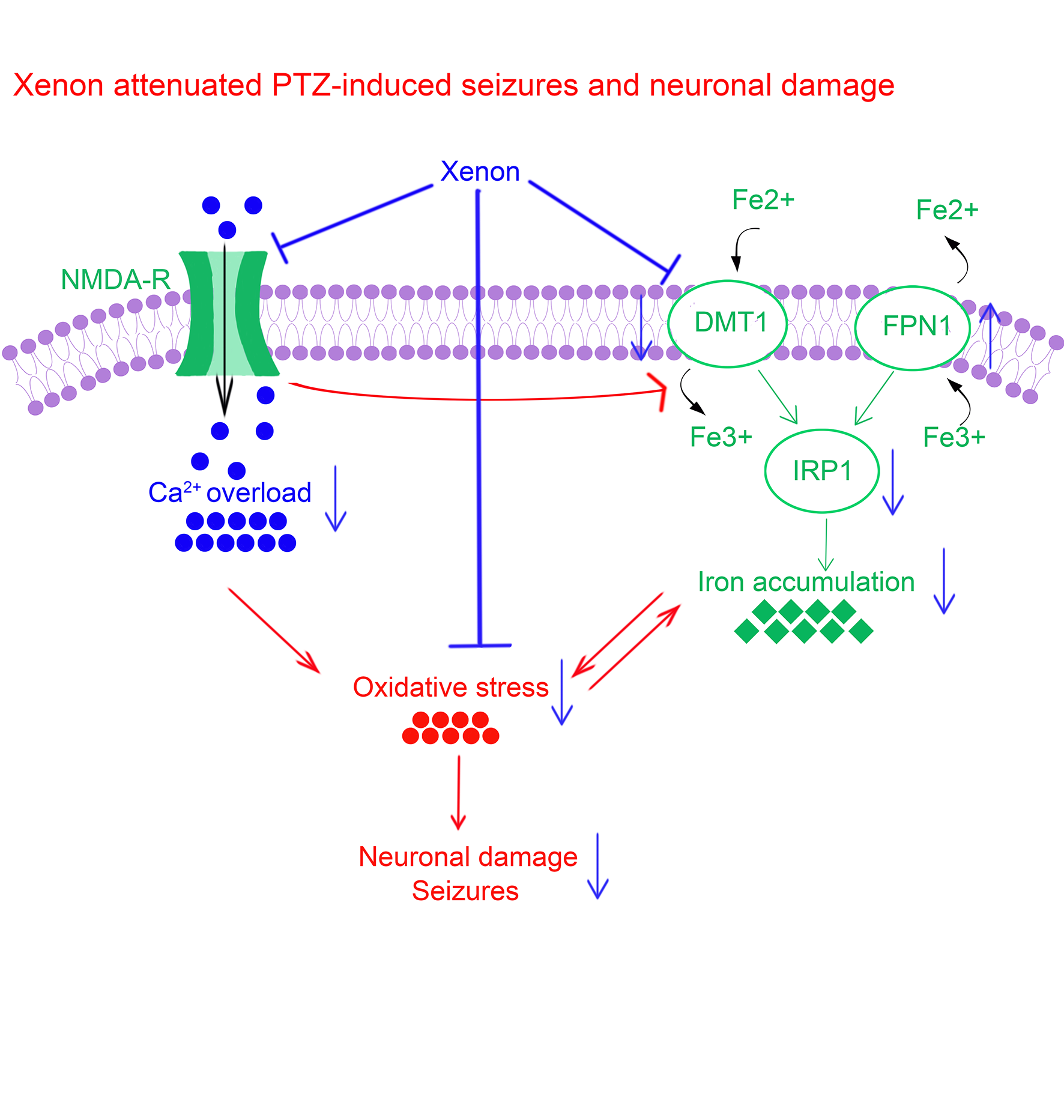

Supplement: Supplementary Figure 5 — The roles and mechanisms of xenon in pentylenetetrazole (PTZ)-induced seizures and neuronal damage. [file Image_5.TIF]
